# Phishing suspiciousness in older and younger adults: The role of executive functioning

**DOI:** 10.1371/journal.pone.0171620

**Published:** 2017-02-03

**Authors:** Brandon E. Gavett, Rui Zhao, Samantha E. John, Cara A. Bussell, Jennifer R. Roberts, Chuan Yue

**Affiliations:** 1 Department of Psychology, University of Colorado Colorado Springs, Colorado Springs, CO, United States of America; 2 Division of Computer Science, Colorado School of Mines, Golden, CO, United States of America; 3 Alzheimer’s Disease Research Center, Department of Neurology, Emory University School of Medicine, Atlanta, GA, United States of America; Waseda University, JAPAN

## Abstract

Phishing is the spoofing of Internet websites or emails aimed at tricking users into entering sensitive information, with such goals as financial or identity theft. The current study sought to determine whether age is associated with increased susceptibility to phishing and whether tests of executive functioning can predict phishing susceptibility. A total of 193 cognitively intact participants, 91 younger adults and 102 older adults, were primarily recruited through a Psychology department undergraduate subject pool and a gerontology research registry, respectively. The Executive Functions Module from the Neuropsychological Assessment Battery and the Iowa Gambling Task were the primary cognitive predictors of reported phishing suspiciousness. Other predictors included age group (older vs. younger), sex, education, race, ethnicity, prior knowledge of phishing, prior susceptibility to phishing, and whether or not browsing behaviors were reportedly different in the laboratory setting versus at home. A logistic regression, which accounted for a 22.7% reduction in error variance compared to the null model and predicted phishing suspiciousness with 73.1% (95% CI [66.0, 80.3]) accuracy, revealed three statistically significant predictors: the main effect of education (*b* = 0.58, *SE* = 0.27) and the interactions of age group with prior awareness of phishing (*b* = 2.31, *SE* = 1.12) and performance on the Neuropsychological Assessment Battery Mazes test (*b* = 0.16, *SE* = 0.07). Whether or not older adults reported being suspicious of the phishing attacks used in this study was partially explained by educational history and prior phishing knowledge. This suggests that simple educational interventions may be effective in reducing phishing vulnerability. Although one test of executive functioning was found useful for identifying those at risk of phishing susceptibility, four tests were not found to be useful; these results speak to the need for more ecologically valid tools in clinical neuropsychology.

## Introduction

Phishing is the spoofing of Internet websites or emails aimed at tricking users into entering sensitive information, such as usernames and passwords [1]. It is one of the most severe and challenging threats to Internet security. As Internet accessibility continues to increase, and as the sophistication of phishing attacks continues to improve [2], individuals of all ages and backgrounds are increasingly susceptible to this threat. Falling victim to a phishing attempt may lead to identity theft and loss of financial resources [3]. In older adults, these outcomes may have important consequences for living independently and for maintaining social, emotional, and medical health.

An abundance of research in the computer sciences and human factors fields has addressed phishing susceptibility and its association with demographic variables. For instance, Metzger et al. [4] reported that students were more likely to trust all information channels and less likely to verify online information compared to nonstudents. Sheng et al. [5] found that women and people between 18 and 25 years old were less suspicious of phishing than people of other ages. The effect of age on phishing susceptibility was largely explained by prior experience with phishing, experience using the Internet, perception of financial risk, and years of education. The average age of their sample, recruited through Amazon’s Mechanical Turk, was 30; but because no other information about the participants’ age was reported, the composition of older adults in that study is unclear [5]. Using a role-play paradigm similar to Sheng and colleagues [5], Welk et al. [6] found that “personality characteristics that support reserved behavior, low impulsivity, and distrust decreased phishing susceptibility within an email-based decision making task” (p. 13). The increased impulsivity that is often characteristic of younger adults has been proposed as another mechanism governing the increased phishing susceptibility reported in this cohort. However, it should be noted that some studies have failed to demonstrate increased susceptibility in the 18–25 age group compared to older groups; for example, Zielinska et al. [7] found no difference between young people (age 18–25) and other people (age ≥ 26) in phishing susceptibility. Nevertheless, no studies of phishing susceptibility to date have focused specifically on older adults (i.e., age 50+).

Similarly, few studies have focused on examining the value of cognitive abilities in predicting phishing susceptibility. Vishwanath and colleagues have reported a series of studies showing that the degree to which participants pay attention to and recall the details of a phishing email are related to whether or not victimization occurs [8–10]. Hong and colleagues [11] measured working memory, crystallized intelligence, spatial skills, and sustained attention in their sample of younger adults, but did not report data related to the association between these cognitive abilities and performance on a phishing detection task. Finally, Purkait et al. [12] found positive associations between phishing detection and experimental measures of attentional vigilance and short-term memory. There are no other studies known to have evaluated the predictive validity of cognitive measures for phishing outcomes.

In the field of clinical neuropsychology, there is a large corpus of research that has focused on the use of cognitive tests to predict real-world functional outcomes, including instrumental activities of daily living (IADLs) such as financial management, driving, and medication adherence [13]. These ecological validity studies are commonly pursued in an older adult population due to the increased risk of aging-related cognitive disorders and their associated functional disabilities [14]. Given the now prevalent use of the Internet and smartphone applications for the organization and management of daily tasks such as shopping, banking, and social networking, predictions about real-world functional outcomes ought to include measures of technology use and its associated risks. The use of technology such as online shopping and banking may represent an enhanced activity of daily living (EADL; [15]) that serves to improve quality of life, whereas one’s ability to resist phishing and other scams may be best conceptualized as an IADL. Despite the threats posed by phishing, one’s ability to recognize and avoid phishing attacks has not been a common focus of research from the perspective of clinical neuropsychology and ecological validity. The current study was designed to bridge this gap in the literature, with the goals of adding a clinical and ecological perspective to the existing security-focused literature on phishing as well as taking the first steps toward promoting the assessment and prediction of phishing susceptibility across age groups.

Current older adult cohorts may be particularly vulnerable to phishing attempts. In fact, older adults appear to be disproportionately targeted for many types of fraud, including financial exploitation [16] as a result of their greater potential for wealth; decreased familiarity, comfort, and self-efficacy using computers; less experience with web browsing [17–20]; and increased risk for age-related cognitive decline [21]. Even in the absence of mild cognitive impairment (MCI) or dementia, older adults may exhibit impairments in decision-making [22]. For example, age-related decline in executive functioning is associated with a decline in functional capacities, such as financial management [23–25]. Research has consistently demonstrated that tests of executive functioning can be useful for the prediction of IADLs, especially in older adults with cognitive impairment due to neurodegenerative diseases [26–29]. For example, the Iowa Gambling Task (IGT) measures decision-making in ambiguous situations, which is believed to be important for performing IADLs [30]. Other research has demonstrated associations between IADL functioning and scores on tests of judgment and decision-making [31,32], maze navigation [33], abstract reasoning and concept formation [34], and verbal fluency [35].

Avoiding online fraud is becoming an important component of one’s capacity to function independently. This is especially true for emerging cohorts of older adults who rely heavily on electronic storage of personal information, as through online banking applications, electronic medical records, and cloud-based storage systems. Clinicians working with older adults with known or suspected cognitive impairment may wish to assess this capacity to predict susceptibility and intervene when appropriate [36]. Recent advances in the conceptualization and assessment of capacity have cautioned against treating this construct as a single entity; instead, it is recommended that capacity be conceptualized as applying to a particular function (e.g., capacity to make financial decisions) [37]. Under this approach, the prediction of phishing susceptibility represents a specific type of capacity that may wish to be addressed to protect vulnerable individuals from financial abuse and identity theft. As current cohorts of digital natives age into older adulthood, assessing the capacity to detect phishing attempts will undoubtedly become an essential focus of clinical assessment.

The current study sought to identify and compare the relationship between cognitive functioning—in particular, executive functioning ability—and phishing susceptibility in younger and older adult samples. Because older adults are generally less familiar with computers [18–20] and are more likely to experience declines in abilities such as judgment and decision-making [21], we hypothesized that the older adult cohort would be more vulnerable to phishing attempts than the younger adult cohort. In addition, we hypothesized that commonly used tests of executive functioning, especially those measuring judgment and decision-making, would be predictive of phishing susceptibility.

## Materials and methods

### Participants

A total of 207 participants were recruited as volunteers for this study. Because we could not use the data from 10 participants due to technological errors and 4 participants for other reasons (confusion, *n* = 2; forgot login information, *n* = 2), the total sample size used in the current analysis was 193. The younger adult sample (*n* = 91) was primarily recruited through the Psychology department’s undergraduate subject pool at the University of Colorado Colorado Springs (UCCS), receiving course credit or extra credit for participation. The older adult sample (*n* = 102) was recruited from the community using a variety of methods, including a University Gerontology Center research registry, targeted newspaper advertisements, flyers, community lectures, and word of mouth. Older adults were compensated $15 for their participation. Inclusion criteria required use of the Internet at least once per month and possession of common Internet website accounts (e.g., Amazon, Google). Exclusion criteria were as follows: traumatic brain injury within the last three months, diagnosis of a major mental illness (e.g., Schizophrenia), use of medications that interfere with cognition, and diagnosis of dementia or other neurological condition. For older adults, the telephone version of the Memory Impairment Screen (MIS-T) [38] was administered to ensure that participants were cognitively intact; participants who scored 5 or lower (cut-off for suspected impairment) on the MIS-T were excluded.

### Procedure

The study received human subjects approval from the University of Colorado Colorado Springs Institutional Review Board. A trained graduate or undergraduate research assistant conducted all data collection in Psychology lab space on campus. At the outset of the study, participants provided written informed consent, answered a demographics questionnaire, and were administered a battery of executive functioning tests (described below). Participants were then asked to perform a series of web-browsing activities, including evaluation of websites’ login notifications, and to then rate their stylistic preferences by site. Participants were not aware that these activities would involve phishing attempts to obtain their login credentials.

Immediately before the web-browsing tasks, the examiner emphasized the voluntary nature of the study to participants and provided reassurance that the tasks could be discontinued at any time without penalty. After the examiner left the room, participants visited four different websites, two of which were legitimate sites and two of which were phishing sites that we created. Of the two total phishing sites, one login utilized traditional methods (i.e., entering one’s username and password for that site) and one utilized single sign-on (SSO) methods (i.e., logging into a website such as Sears.com using an identity provider account, such as Facebook or Google). The order of the legitimate and phishing websites, and the traditional vs. SSO websites, was counterbalanced. Participants’ interactions with these sites were monitored remotely to determine whether and where login attempts were made. No sensitive login information was recorded, transmitted, or stored when participants interacted with the phishing websites.

**Fig 1:**
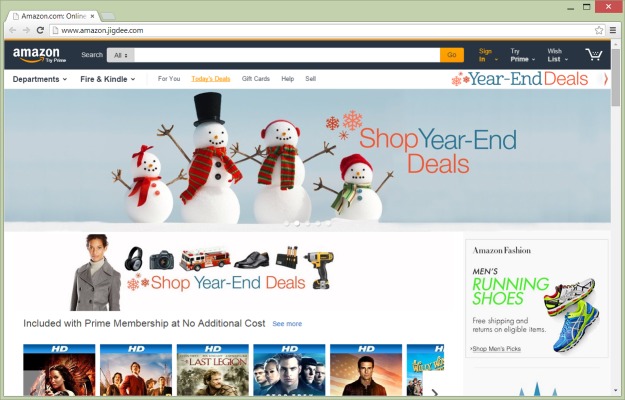
Example of a Phishing Home Page Encountered by Study Participants. A dynamically generated homepage on the Amazon phishing website (it presents the same content that is actually displayed on the legitimate Amazon website).

**Fig 2:**
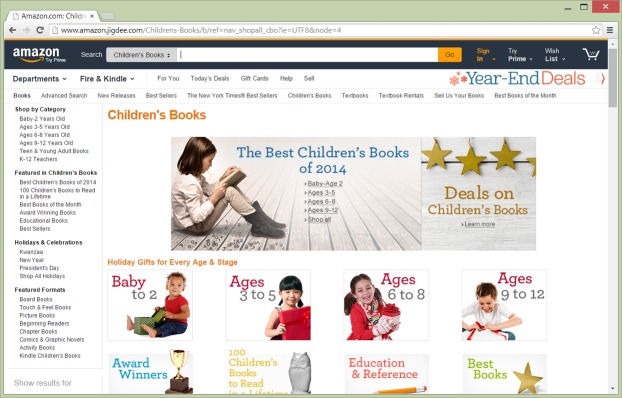
Example of a Deep-Level Phishing Web Page Encountered by Study Participants. A dynamically generated deep-level webpage on the Amazon phishing website (any level of webpages can be generated and presented to users in real-time).

Phishing websites were hosted on university servers and designed to accurately mimic the target websites, so that activities such as clicking on links, searching, and logging in would behave identically to target websites. URLs of the phishing websites were created with the explicit purpose of alerting participants to the forgery site (e.g., http://www.amazon.jigdee.com instead of http://www.amazon.com). Figs 1 and 2 present examples of phishing websites used in this study. Fig 1 shows a screenshot of the phishing testbed’s replica of the Amazon.com homepage and Fig 2 shows a dynamically generated deep-level replica of the Amazon.com website. A more complete description of the phishing testbed has been published previously [1]. The phishing testbed can also be accessed through our project website, hosted at http://inside.mines.edu/~chuanyue/NSF/NSF1624149.html.

After participants completed the web-browsing task, the examiner returned to the room and administered several questionnaires about the web-browsing activity without disclosing the phishing attempts. The questionnaires asked whether the participants noticed anything suspicious about the websites, whether their approach to the web-browsing task would have differed if they were at home rather than in a lab, whether they had prior knowledge of phishing, and whether they had previously been victimized by phishing. A copy of the questionnaire is included as S1 Appendix. After the completion of these questionnaires, participants were debriefed about the true purpose of the study and compensated for their time.

### Neuropsychological measures

#### Neuropsychological Assessment Battery (NAB) executive functions module

The NAB Executive Functions Module [39] contains four tests that measure an array of abilities related to executive functioning. The scores on these four tests can be combined to produce a demographically corrected (age, sex, and education) Executive Functions Index Score (*M* = 100, *SD* = 15) that characterizes global executive functioning abilities. On all NAB tests, higher scores reflect better performance.

NAB Mazes. The NAB Mazes test (range = 0–26) requires participants to complete up to seven pen-and-paper mazes that increase in difficulty. Points are earned for completing the mazes within the allotted time, with more points awarded for more rapid completion times.

NAB Judgment. The NAB Judgment test (range = 0–20) asks participants to answer 10 questions about health and safety situations and has demonstrated ecological validity for various decision-making tasks [31,32].

NAB Categories. The NAB Categories test presents participants with pictures of six fictional people, along with details about each person. The participant is asked to classify the six-pictured individuals into two characteristically based groups of two to four people according to information presented. Groupings can be based on a variety of concrete and abstract features. The test consists of two trials, with identical rules but different pictured individuals. Accurate groupings are awarded points from 0–2.

NAB Word Generation. The NAB Word Generation test asks participants to generate as many three-letter words as possible from a pictured series of seven letters. Correct words are awarded 1 point.

#### Iowa Gambling Task (IGT)

The IGT [40] asks participants to imagine they are loaned money to play a card game, where each card picked from one of four decks causes them to win or lose money. Participants are instructed to win as much money as possible, but are not warned that the decks possess distinct patterns and systematic differences in credits and debits. Higher Net Total raw scores reflect the prioritization of better decks (and better decision-making) when picking cards. Past research has supported the relationship between low IGT scores, poor decision-making, and ventromedial prefrontal cortex dysfunction [41,42].

### Data analysis

All analyses were performed in R version 3.3.2 [43]. An a priori alpha level was set at .05. The study’s primary hypothesis was tested with logistic regression via the *lrm* function in the *rms* package version 5.0–1 [44]. Missing data were handled using multiple imputation with five imputed data sets. Initially, phishing susceptibility was intended to be the primary dependent variable, but a lack of variance in this outcome was found (only 12 participants declined to login to the phishing websites). Therefore, the dependent variable chosen instead was whether or not participants reported feeling suspicious of one or more websites during the browsing activities.

Suspiciousness (yes/no) was modeled as a function of the following predictor variables: Prior knowledge of phishing (yes/no); prior susceptibility to phishing (yes/no); lab vs. home differences in approach (yes/no); education (years); sex (male/female); ethnicity (Hispanic/not Hispanic); race (Caucasian/not Caucasian); NAB Mazes, Judgment, Categories, and Word Generation (raw scores); and IGT Net Total (raw score). In addition to these main effects, the interactions between these variables and Age Group (younger/older) were also modeled to investigate if age group moderates the ability of predictor variables to explain phishing suspiciousness.

## Results

Participant demographic data, cognitive test results, and answers to phishing questionnaires are presented in Table 1. On the IGT, three participants admitted and two demonstrated (through perfect or nearly perfect scores) prior knowledge of the task; therefore, we omitted IGT scores for these five participants. As described above, only 12 were not vulnerable to the experimental phishing attacks. All 12 participants who chose not to enter their login information to the phishing sites were older adults. Of these 12 older adult participants, 10 reported prior knowledge of phishing and 4 reported being victims of phishing in the past; their mean age was 69.17 (*SD* = 9.4) and their mean years of education was 16.5 (*SD* = 2.11). Compared to the number of participants who were victimized by the experimental phishing attacks in the current study, there was more variability about reported suspiciousness, with 68 participants reporting something suspicious about one or more of the websites.

**Table 1:** Participant Demographics, Cognitive Test Scores, and Questionnaire Responses.

Variable	Total	Younger	Older
N	193	91	102
Mean Age, years (SD) [range]*	45.70 (22.63) [18, 88]	22.90 (5.90) [18, 44]	66.05 (7.50) [50, 88]
Mean Education, years (SD) [range]*	14.93 (1.98) [12, 20]	13.8 (1.20) [12, 16]	15.94 (2.00) [12, 20]
Female Sex, N (%)	134 (69.79%)	62 (68.13%)	72 (71.29%)
Caucasian Race, N (%)*	154 (82.35%)	63 (71.59%)	91 (91.92%)
Hispanic Ethnicity, N (%)*	34 (18.09%)	28 (30.77%)	6 (6.19%)
First Language English, N (%)	181 (95.26%)	83 (92.22%)	98 (98.00%)
NAB EF Index, M (SD) [range]*	106.17 (17.70) [64, 151]	98.12 (12.68) [65, 125]	113.35 (18.49) [64, 151]
NAB Mazes, M (SD) [range]*	16.99 (6.66) [2, 26]	20.25 (4.88) [7, 26]	14.09 (6.71) [2, 26]
NAB Judgment, M (SD) [range]	15.37 (2.66) [6, 20]	15.32 (2.33) [7, 20]	15.42 (2.94) [6, 20]
NAB Categories, M (SD) [range]*	27.78 (9.57) [3, 53]	29.63 (9.01) [9, 53]	26.13 (9.80) [3, 51]
NAB Word Generation M (SD) [range]*	11.82 (4.72) [2, 28]	10.78 (4.02) [2, 22]	12.75 (5.10) [3, 28]
IGT Net Total, M (SD) [range]	19.11 (27.00) [–54, 72]	17.84 (25.74) [–54, 62]	20.18 (28.09) [–44, 72]
Suspicious of Websites, N (%)	68 (35.23%)	26 (28.57%)	42 (41.18%)
Knowledge of Phishing, N (%)*	155 (80.31%)	65 (71.43%)	90 (88.24%)
Past Victim of Phishing, N (%)*	78 (40.62%)	24 (26.37%)	54 (53.47%)
Reported Lab vs. Home Difference, N (%)*	64 (33.68%)	17 (18.68%)	47 (47.47%)

NAB = Neuropsychological Assessment Battery; IGT = Iowa Gambling Task.

* Significant difference between younger and older adults (*p* < .05).

Overall, the younger adult group contained a greater proportion of individuals of non-Caucasian race, *χ*^2^ (*df* = 1) = 11.89, *p* = 0.001, and Hispanic/Latino/Spanish ethnicity, *χ*^2^ (*df* = 1) = 17.53, *p* < 0.001. Older adults had more years of education, *t* (*df* = 168.32) = -9.10, *p* < 0.001. The younger adult group performed better on NAB Mazes, *t* (*df* = 183.67) = 7.35, *p* < 0.001, and NAB Categories, *t* (*df* = 190.82) = 2.58, *p* = 0.011, while the older adult group demonstrated better overall executive functioning on the demographically-corrected NAB Executive Functions Index score, *t* (*df* = 179.58) = -6.73, *p* < 0.001, and produced higher scores on the NAB Word Generation test, *t* (*df* = 188.19) = -2.99, *p* = 0.003. More older adults than younger adults reported knowledge of phishing, *χ*^2^ (*df* = 1) = 7.56, *p* = 0.006. They were also more likely to have been victimized by phishing in the past, *χ*^2^ (*df* = 1) = 13.46, *p* < 0.001, and more likely to report that their web-browsing behaviors were different in the laboratory setting versus at home, *χ*^2^ (*df* = 1) = 16.33, *p* < 0.001. No other group differences were observed on any of the variables reported in Table 1.

**Fig 3:**
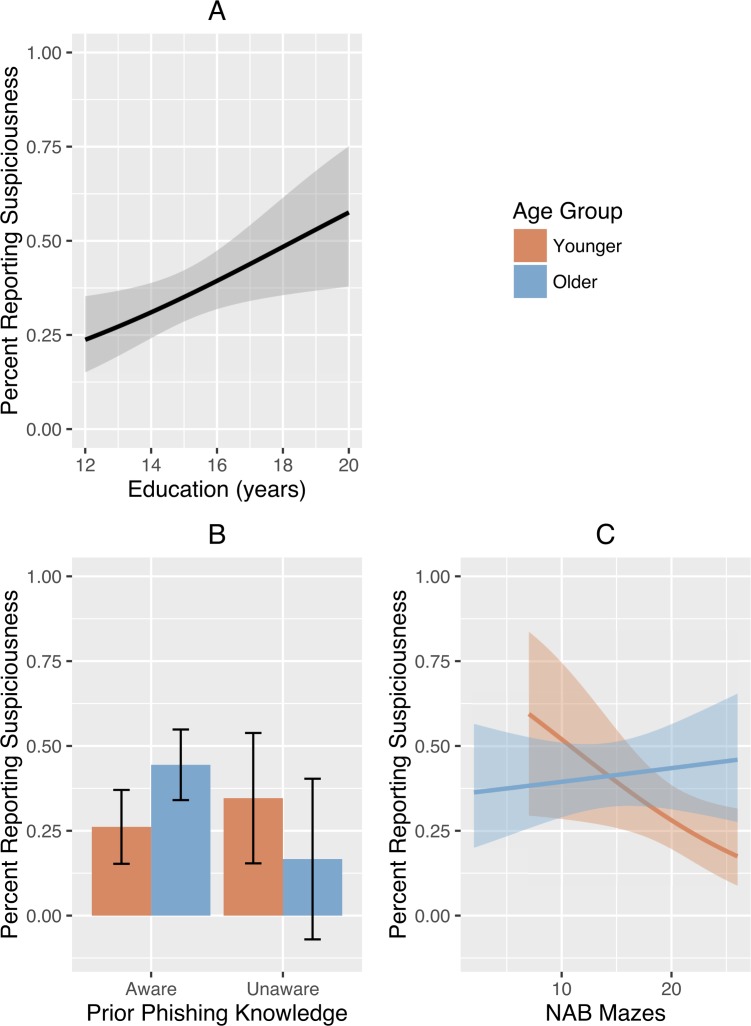
Predictors of Phishing Susceptibility Derived from Logistic Regression. Main effect of years of education on reported phishing suspiciousness (A). Age group interaction effects with prior phishing knowledge (B) and NAB Mazes score (C). Error bars and shaded bands represent 95% confidence intervals.

The results of the logistic regression demonstrated that the predictor variables contributed to a 22.7% advantage for the hypothesized model over an intercept-only model (Nagelkerke *R*^2^), *χ*^2^ (*df* = 25) = 34.85, *p* = 0.092. Detailed results of the logistic regression model are presented in Table 2. As can be seen from Table 2, there were two significant main effects (years of education and NAB Mazes raw score); in addition, prior knowledge of phishing and scores on NAB Mazes were significantly moderated by Age Group. These effects are depicted graphically in Fig 3. The model correctly classified participants’ phishing suspiciousness with 73.1% (95% CI [66.0, 80.3]) accuracy.

**Table 2:** Results of the Logistic Regression Analysis Predicting Phishing Suspiciousness.

Predictor	*b*	SE	95% CI_*b*_	*z*	*p*	OR	95% CI_*OR*_
Intercept	-5.46	4.11	[-13.51, 2.59]	-1.33	.184	0.00	[0.00, 13.37]
Main Effects							
Age Group	1.31	4.78	[-8.07, 10.68]	0.27	.785	3.69	[0.00, 43655.35]
Prior Knowledge	-0.85	0.71	[-2.23, 0.54]	-1.20	.230	0.43	[0.11, 1.71]
Past Susceptibility	-0.05	0.69	[-1.41, 1.31]	-0.08	.938	0.95	[0.24, 3.69]
Lab vs. Home	1.07	0.68	[-0.26, 2.41]	1.58	.115	2.92	[0.77, 11.10]
Education	0.58*	0.27	[0.05, 1.10]	2.16	.031	1.78	[1.05, 3.01]
Sex	0.06	0.65	[-1.20, 1.33]	0.10	.922	1.06	[0.30, 3.77]
Ethnicity	-0.40	0.65	[-1.69, 0.88]	-0.62	.538	0.67	[0.19, 2.41]
Race	-0.97	0.72	[-2.39, 0.45]	-1.34	.180	0.38	[0.09, 1.56]
NAB Mazes	-0.14*	0.06	[-0.25, -0.02]	-2.33	.020	0.87	[0.78, 0.98]
NAB Judgment	0.05	0.12	[-0.19, 0.29]	0.42	.676	1.05	[0.83, 1.34]
NAB Categories	-0.01	0.04	[-0.08, 0.05]	-0.40	.687	0.99	[0.92, 1.06]
NAB Word Generation	-0.01	0.08	[-0.17, 0.15]	-0.11	.912	0.99	[0.84, 1.17]
IGT Net Total	-0.02*	0.01	[-0.05, 0.00]	-2.02	.044	0.98	[0.96, 1.00]
Interaction Effects							
AG x PK	2.31*	1.12	[0.11, 4.51]	2.05	.040	10.07	[1.11, 91.17]
AG x PS	0.29	0.84	[-1.36, 1.94]	0.34	.732	1.33	[0.26, 6.94]
AG x LH	-0.99	0.82	[-2.60, 0.63]	-1.20	.231	0.37	[0.07, 1.88]
AG x Education	-0.49	0.29	[-1.06, 0.09]	-1.66	.096	0.62	[0.35, 1.09]
AG x Sex	-0.48	0.82	[-2.08, 1.13]	-0.58	.562	0.62	[0.12, 3.10]
AG x Ethnicity	1.29	1.23	[-1.11, 3.70]	1.05	.291	3.65	[0.33, 40.34]
AG x Race	1.71	1.10	[-0.45, 3.87]	1.55	.122	5.52	[0.63, 48.06]
AG x NAB Mazes	0.16*	0.07	[0.02, 0.29]	2.30	.022	1.17	[1.02, 1.34]
AG x NAB Judgment	-0.06	0.15	[-0.35, 0.23]	-0.41	.679	0.94	[0.70, 1.26]
AG x NAB Categories	0.00	0.05	[-0.09, 0.09]	0.05	.961	1.00	[0.92, 1.10]
AG x NAB Word Generation	0.09	0.10	[-0.10, 0.28]	0.89	.373	1.09	[0.90, 1.32]
AG x IGT Net Total	0.02	0.01	[-0.01, 0.05]	1.51	.131	1.02	[0.99, 1.05]

OR = Odds ratio; NAB = Neuropsychological Assessment Battery; IGT = Iowa Gambling Task; AG = Age Group; PK = Prior Knowledge; PS = Past Susceptibility; LH = Lab vs. Home. For categorical predictor variables, reference groups are younger (Age Group), no prior knowledge of phishing (Prior Knowledge), no past phishing susceptibility (Past Susceptibility), no difference between lab and home (Lab vs. Home), female (Sex), non-Hispanic (Ethnicity), and Caucasian (Race).

* *p* < .05.

## Discussion

This study sought to determine whether older adults are more susceptible to computer-based phishing attacks and whether cognitive test scores can predict phishing susceptibility. Because very few participants declined to log in to the experimental phishing websites, we used reported suspiciousness as our dependent variable. Overall, the logistic regression model was able to achieve a classification accuracy of 73.1% (95% CI [66.0, 80.3]) in predicting whether or not participants would be suspicious of the experimental phishing attacks. The results presented here failed to support our hypothesis that older adults would be more susceptible to phishing than younger adults. Although the results indicate that being an older adult was associated with 3.69 times greater odds of being suspicious relative to younger adults, this parameter was estimated too imprecisely to interpret with confidence (Table 2). There was partial support for our hypothesis that executive functioning tests would be predictive of phishing susceptibility. One test, NAB Mazes, was predictive of phishing susceptibility. Other executive functioning test scores were not important predictors of phishing susceptibility. More specific findings are discussed below.

Compared to younger adults, older adults were more likely to have knowledge of phishing and more likely to have been victimized by phishing in the past. In addition, older adults appeared to have been less trusting of the research setting, as they were more likely to report using different browsing behaviors in the lab versus at home, and all 12 participants who resisted the phishing attacks were older adults. Older adults’ suspiciousness varied markedly depending on whether or not they possessed prior knowledge of phishing. When broken down by prior knowledge (Fig 3B), the most suspicious of the subgroups was the older adults with prior knowledge of phishing, whereas the least suspicious subgroup was the older adults without prior knowledge of phishing. In contrast, younger adults without prior knowledge of phishing reported more suspiciousness than those with prior knowledge. Because all of the younger adults—whether suspicious or not—entered their login information into phishing sites, this suggests that younger adults may have been too trusting of the laboratory environment. Consistent with the findings of Metzger and colleagues [4], our younger adult participants, all of whom were students at the university where this study was performed, trusted the experimental setting and the website content. For older adults, personal experience (having been victimized by phishing) and associated prior knowledge may have offered useful protection against fraud. For younger adults, experience and information may have had the opposite effect by creating implicit trust in the research setting, which likely includes trust about IT security. Also as expected, people with more years of education tended to be more suspicious of the phishing attempts (Table 2), which is consistent with previous research suggesting that years of education moderates the influence of age group on susceptibility to phishing [5]. It is likely that educational background is related to factors associated with learning and cognition, but it may also serve as a proxy for experience and familiarity with computers, as has been previously reported [17]. Unlike other studies (e.g., [5,11,45]), we found no differences in phishing suspiciousness between men and women.

Only one cognitive test, NAB Mazes, was strongly associated with phishing suspiciousness. The interaction between this variable and age group produced an unexpected pattern, however. In older adults, better test scores were associated with more suspiciousness, as expected. But in younger adults, better test scores were associated with less suspiciousness (Fig 3). The best explanation for this pattern may be that the abilities measured by NAB Mazes (processing speed, planning) serve different roles for younger and older adults: for younger adults, these executive functions contribute to efficient, automatic processing, especially in situations that are perceived as trustworthy (e.g., using campus computers); for older adults, these executive functions may contribute to more deliberative, self-protecting behaviors, especially when they are knowledgeable about risks (e.g., phishing and web browsing) [46]. Similarly, it may be the case that the NAB Mazes test measures slightly different abilities in older versus younger adults. For example, in older adults, the NAB Mazes test may draw more heavily on skills such as visual scanning and spatial navigation; older adults who are better at these skills may be more adept at noticing webpage anomalies that increase suspiciousness. In younger adults, in contrast, the NAB Mazes test may draw more heavily on information processing speed and mental efficiency; younger adults who are better at these skills may have used cognitive shortcuts, such as the familiarity heuristic, which could have suppressed detection of webpage anomalies and reduced suspiciousness [47]. The possibility that the NAB Mazes test draws on different cognitive abilities in younger and older adults suggests measurement invariance, which should be a focus of future research on this test and the rest of the NAB Executive Functions module.

In contrast to the effects discussed above, statistical significance was not reached for several predictor variables, including past phishing susceptibility, lab versus home browsing approach, sex, race, ethnicity, three tests from the NAB Executive Functions module, and the IGT. These results suggest that most demographic variables, including age cohort, are insufficient predictors of phishing victimization. Although race and ethnicity were associated with some of the larger effect sizes (Table 2), these parameters were estimated extremely imprecisely due to the limited representation of ethnic and racial minorities in the current sample, especially among older adults (Table 1). Examining phishing susceptibility differences across ethnic and racial groups is important, as evidence suggests that these groups report different Internet usage patterns [17]. These results also suggest that some tests of executive functioning are limited in their utility to identify potential victims of phishing and possibly other forms of exploitation. It should be pointed out, however, that both the main and interaction effects of the IGT were very close to reaching statistical significance (Table 2). Nevertheless, these parameters were estimated with good precision, suggesting that the ability of IGT scores to predict phishing suspiciousness is of limited clinical significance regardless of statistical significance. Taken together, the predictor variables used here were able to predict—with an accuracy of 73.1% (95% CI [66.0, 80.3])—whether or not an individual participant would report suspiciousness. However, because the logistic regression model only produced a 22.7% reduction in error variance compared to the null model, it is likely that there are additional unmeasured variables that could greatly contribute to the prediction of phishing suspiciousness and susceptibility. The current results are best interpreted as the first step in identifying ecologically valid neuropsychological approaches for identifying phishing susceptibility and are not suited for classification purposes at the individual level. Because the current results have not been cross-validated or validated in out-of-set data, additional studies are needed before using neuropsychological tests to classify individuals based on predicted phishing susceptibility.

Neuropsychological assessment instruments have been described as having limited ecological validity [48,49]. The results of the current study also suggest considerable room for improvement in the use of neuropsychological tests for the prediction of real-world functional outcomes. Neuropsychologists are often asked to evaluate individuals to render judgments about functional capacities such as financial management and ability to avoid exploitation [50]. Because phishing represents an emerging threat to individuals lacking in those capacities, it is necessary for neuropsychologists to have access to clinical tools that are valid for making such judgments. These results highlight the importance of continued development of assessment techniques that can jointly measure cognitive and functional abilities and that are predictive of real-world outcomes. Cognitive abilities have been shown to play an important role in older adults’ ability to learn and adapt to advances in computing technology [17], which suggests that the development of novel neuropsychological tests that can better predict computer-related EADLs and IADLs is of considerable importance.

The current study is limited by the salient influence of the laboratory environment. Out of the 29 individuals who reported prior knowledge of phishing, prior victimization by phishing, and suspiciousness of the websites visited, 26 (89.7%) entered their login information into the spoofed web pages. We attempted to minimize the influence of the laboratory setting by emphasizing that participants could discontinue any task without penalty, by having examiners leave the room while participants browsed the Internet, and by asking participants to enter their private login data into real (or seemingly real) websites, some of which may have been used to store sensitive data (e.g., credit cards). This aspect of the study represents a strength relative to other research, which often asks participants to indicate how they might respond to a role-play scenario (e.g., [5,45]) or to simply identify whether a website is legitimate (e.g., [11]). However, it appears as though the powerful influences of trust, obedience, and authoritarianism affected our participants [51]. Past research has implicated trust as an important factor influencing phishing susceptibility [52], and current methods used by websites to demonstrate their own trustworthiness are of marginal effectiveness [53]. However, because so few participants were able to avoid becoming susceptible to phishing, our resulting shift in focus from susceptibility to suspiciousness may be capturing other non-specific personality traits, such as skepticism, paranoia, or anxiety, that were not directly related to the phishing attempts [17,54]. Further, our study may have been improved through the inclusion of additional cognitive predictors of phishing suspiciousness, such as more basic aspects of attention, which have been proposed as a mechanism for phishing susceptibility in the past (e.g., [9]). However, given the fundamental role of attention skills in completing more complex tasks of executive functioning, it is likely that individual variability in attention skills was captured indirectly through the battery of executive functioning tests used here [55].

This study is also limited by the fact that our older adult sample was self-selected to participate in this study and is therefore unlikely to have good external validity for the entire population of Internet-using older adults, or older adults who use the internet less frequently than the minimum of once per month needed to meet our study’s inclusion criteria. Our older adult sample was highly educated, in possession of higher than average executive functioning abilities, and lacking in racial and ethnic diversity. Approximately 88% of the older adult sample reported knowledge of phishing (even more than the 71% reported in younger adults), which may be higher than in the general population of Internet-using older adults. Because almost 53% of the older adults in our sample reported past victimization by phishing attacks, it is possible that this high rate of phishing knowledge in older adults is caused by high rates of past victimization. It should also be mentioned that our study’s operationalization of younger and older age cohorts may affect the results. For example, some studies have used age 65 as the cutoff for defining "older" adults. Similarly, two individuals older than 40 were included in our younger adult group. Additional limitations include the fact that some parameters were not estimated with desirable precision (Table 2), and the fact that we did not ask participants about their familiarity with the target websites. Although we ensured that every phishing webpage was based on a website at which participants owned a real user account, participants may have differed in the frequency with which they used these sites. Finally, we excluded participants with cognitive impairment, so our results cannot speak to the risk of phishing susceptibility in older adults with MCI or dementia. Future research should evaluate susceptibility to phishing in individuals with MCI, as this group will continue to grow in size with the emerging baby-boomer cohort, many of whom are avid or frequent technology users.

Many phishing attempts are predicated upon trust and not necessarily technological sophistication. As such, the current results suggest that both older and younger adults can be protected from phishing attempts through methods that provoke discomfort and suspiciousness at the expense of automatic processing. Educational initiatives that increase phishing awareness while also directly linking phishing threats to concrete negative consequences may provide the necessary skills to avoid being phished. Past research supports the use of education as a strategy for intervention and means for decreasing vulnerability; similar initiatives have shown promise in increasing knowledge and suspiciousness about phishing in both older and younger adults. A training intervention employed by Sheng and colleagues showed that phishing education could help reduce users’ tendency to become victims [5]. Carpenter et al. reported that warnings are effective at reducing online personal information disclosure [56]. Kumaraguru et al. developed an email-based anti-phishing education system, PhishGuru, and an online game, Anti-Phishing Phil, that uses learning science instructional principles to teach users how to use cues in URLs to avoid falling into phishing attacks. Their results suggested that an education approach facilitated the identification of phishing emails and websites [57]. In addition to the above, future research should attempt to investigate phishing susceptibility outside of a laboratory environment where the presence of implicit trust, especially on the part of students, may be influencing behaviors and decreasing the external validity of the results.

In conclusion, the results of this study reveal that older and younger adults both become suspicious about phishing at approximately equal rates, a previously understudied effect within older adults, but that the factors driving phishing susceptibility have similarities and differences between these two age cohorts. For both groups, more years of education appears to be protective against phishing susceptibility. In older adults, lack of knowledge about phishing and lower scores on NAB Mazes contributed to reduced suspiciousness and, presumably, greater susceptibility to phishing. These results also suggest an increased need for development of ecologically valid assessment instruments to identify individuals who are most vulnerable to phishing and similar exploits [49,50]. As access to technology continues to increase worldwide, the potential for victimization by this technology will also increase. Neuropsychologists and other clinicians who wish to identify individuals lacking the capacity to avoid this victimization are in need of more ecologically valid tools than those currently available.

## Supporting information

S1 Appendix Trustworthiness Questionnaire. Administered to participants after the web browsing activity and before the experimental phishing attempts were disclosed.
(PDF)
