# Supplementary material for: Phishing suspiciousness in older and younger adults: The role of executive functioning
Source: PLoS One. 2017 Feb 3;12(2):e0171620. doi: 10.1371/journal.pone.0171620 (PMC5291531; doi:10.1371/journal.pone.0171620)
Supplement: S1 Appendix — Administered to participants after the web browsing activity and before the experimental phishing attempts were disclosed. (PDF) [file pone.0171620.s001.pdf]

Participant ID: ________________ Date: ________________ Examiner: ____________________

Please answer the following questions about your experience with the web browsing activities:

1. Had you heard of "Web Single Sign-On" before coming in today?

   Yes No

2. Did you notice anything suspicious about the websites that you visited during this study?

   Yes No

   Please explain your answer to this question from the following perspectives:

   (1) Website look and feel:

   ____

   (2) Website URL address:

   ____

   (3) Website identity certificate:

   ____

   (4) Other (please specify):

   ____

3. If you signed into any of the websites during this study, did you use your correct username and password?

   Yes No

4. Being that you are in a laboratory environment, would you have reacted differently to any of the websites on your computer at home?

   Yes No

5. What makes a website seem "trustworthy" to you? (circle all that apply)

   Website look and feel   Website URL address   Website identity certificate

   Other (please specify):____

6. Are you aware of phishing attacks?

   Yes   No

7. Have you been susceptible to any phishing attacks in the past?

   Yes   No

8. Do you prefer to create a dedicated account for a website to sign into it or do you prefer to sign into the website using your Google, Facebook, or Yahoo account?

   a. Creating and using a dedicated account
   b. Using my Google, Facebook, or Yahoo account

   Please explain your answer to this question:

   ____

   ____

   ____

9. Security is a concern when I perform web browsing activities.
   a. Strongly Disagree
   b. Slightly Disagree
   c. Neutral
   d. Slightly Agree
   e. Strongly Agree

   Please explain your answer to this question:

   ____

   ____

   ____

10. What security measures, if any, do you use to protect yourself from online identity fraud when using the Internet on your own computer?

____

____

____

11. If you have any comments or questions about this study, please write them down:

____

____

____

Thank you for taking part in this study. Your time is greatly appreciated.
